# Identification of Requirements for a Postoperative Pediatric Pain Risk Communication Tool: Focus Group Study With Clinicians and Family Members

**DOI:** 10.2196/37353

**Published:** 2022-07-15

**Authors:** Michael D Wood, Kim Correa, Peijia Ding, Rama Sreepada, Kent C Loftsgard, Isabel Jordan, Nicholas C West, Simon D Whyte, Elodie Portales-Casamar, Matthias Görges

**Affiliations:** 1 Department of Anesthesiology Pharmacology and Therapeutics The University of British Columbia Vancouver, BC Canada; 2 BC Children’s Hospital Research Institute Vancouver, BC Canada; 3 School of Information The University of British Columbia Vancouver, BC Canada; 4 Patient Partner Vancouver, BC Canada; 5 Department of Pediatrics The University of British Columbia Vancouver, BC Canada

**Keywords:** eHealth, risk communication, risk, decision aid, pain, individualized risk, surgery, anesthesia, anesthetic, anesthesiology, focus group, requirement definition, prototyping, prototype, pediatrics, pediatric, child, postoperative, prediction, digital health, development, user feedback, patient feedback, user-centered design

## Abstract

**Background:**

Pediatric surgery is associated with a risk of postoperative pain that can impact the family’s quality of life. Although some risk factors for postoperative pain are known, these are often not consistently communicated to families. In addition, although tools for risk communication exist in other domains, none are tailored to pediatric surgery.

**Objective:**

As part of a larger project to develop pain risk prediction tools, we aimed to design an easy-to-use tool to effectively communicate a child’s risk of postoperative pain to both clinicians and family members.

**Methods:**

With research ethics board approval, we conducted virtual focus groups (~1 hour each) comprising clinicians and family members (people with lived surgical experience and parents of children who had recently undergone surgery/medical procedures) at a tertiary pediatric hospital to understand and evaluate potential design approaches and strategies for effectively communicating and visualizing postoperative pain risk. Data were analyzed thematically to generate design requirements and to inform iterative prototype development.

**Results:**

In total, 19 participants (clinicians: n=10, 53%; family members: n=9, 47%) attended 6 focus group sessions. Participants indicated that risk was typically communicated verbally by clinicians to patients and their families, with severity indicated using a descriptive or a numerical representation or both, which would only occasionally be contextualized. Participants indicated that risk communication tools were seldom used but that families would benefit from risk information, time to reflect on the information, and follow-up with questions. In addition, 9 key design requirements and feature considerations for effective risk communication were identified: (1) present risk information clearly and with contextualization, (2) quantify the risk and contextualize it, (3) include checklists for preoperative family preparation, (4) provide risk information digitally to facilitate recall and sharing, (5) query the family’s understanding to ensure comprehension of risk, (6) present the risk score using multimodal formats, (7) use color coding that is nonthreatening and avoids limitations with color blindness, (8) present the most significant factors contributing to the risk prediction, and (9) provide risk mitigation strategies to potentially decrease the patient’s level of risk.

**Conclusions:**

Key design requirements for a pediatric postoperative pain risk visualization tool were established and guided the development of an initial prototype. Implementing a risk communication tool into clinical practice has the potential to bridge existing gaps in the accessibility, utilization, and comprehension of personalized risk information between health care professionals and family members. Future iterative codesign and clinical evaluation of this risk communication tool are needed to confirm its utility in practice.

## Introduction

### Background

Approximately 1 in 5 children experiences persistent postoperative pain at 12 months following surgery [1], which can substantially impact their quality of life, opioid consumption, frequency of hospital visits, and overall trust in the health care system [2]. Thus, improving pediatric pain management [3] using a patient-centered approach [4] has become a strategic priority at BC Children’s Hospital (BCCH, where BC stands for British Columbia). Some risk factors for pediatric postoperative pain have been previously identified (eg, anxiety, poor pain coping skills, and pain catastrophizing) [1,5-7]. In contrast, providing prehabilitation plans outlining patient-specific interventions (eg, diet and nutritional supplementation [8,9] and improving physical function and exercise capacity [9-12]) have resulted in improved postoperative outcomes (eg, decreased length of stay [13] and reduced pain [14]). Combining identified risk factors for postoperative pain and tailored interventions provides opportunities to improve pediatric pain management to optimize postoperative outcomes.

The use of risk communication tools prior to surgery can be engaging and may result in the majority of patients understanding their surgery-associated risks well [15,16]. Several surgical risk stratification scores/tools have been developed to provide risk estimates with the goal of informing and improving care [17]. Although preliminary, these tools have enhanced patient risk comprehension, perceived quality of preoperative clinical conversations, and physician prognostic accuracy, and there is evidence they can decrease length of hospital stay [18]. However, a recent scoping review identified that only 7 (<1%) of 796 screened studies both described the methods used to calculate personalized risk and communicated these findings directly to the patient or health care professional or both [18], and many tools have failed to include patient-centered design principles [15,19]. Finally, risk communication tools should apply best practices when communicating information to patients, including the use of plain language and pictographs to present information visually [20].

Currently, there are no best practices for designing risk communication technologies for use within complex clinical settings. As such, tool/score development would benefit from applying patient-oriented research methods [21-23], user-centered design principles [24], and human factor engineering methods [24-27] to elicit design requirements that effectively communicate procedure-associated risks to both families and clinicians.

### Objectives

Our long-term goal is to reduce the incidence of postoperative pain and long-term opioid use by developing a risk prediction tool, which will generate risk scores from health care data using machine learning techniques, to guide clinicians and family members in informed and collaborative decision-making to reduce these risks or mitigate their effects. The purpose of this study is to define the requirements and features for a potential prototype risk visualization and communication tool by conducting focus groups with our expected end users (ie, parents and clinicians) and to apply human-centered design principles to generate an initial prototype.

## Methods

### Study Design

We conducted a semistructured qualitative study with a convenience sample comprising parents of children who had previously undergone surgery, adults with lived pediatric surgical experience, and clinicians (ie, attending physicians and nurse practitioners) who work at the BCCH.

### Ethical Considerations

Ethical approval was obtained from the Children’s & Women’s Health Centre of British Columbia Research Ethics Board, University of British Columbia (H20-00613; date of approval October 20, 2020; principal investigator [PI] M. Görges). Our findings are reported in accordance with the Consolidated Criteria for Reporting Qualitative Research [28].

### Participants

Clinicians were approached via departmental email distribution lists. Parents were recruited through email lists obtained from the BCCH patient experience office, as well as in person in the Anesthetic Care Unit during their child’s hospital visit. Adults with previous childhood surgery were recruited via provincial research networks (ie, Reach BC and the BCCH patient experience office e-network). After a trained research team member described the study in detail, informed consent was obtained by research staff in person or virtually, with electronic consent documented using Research Electronic Data Capture (REDCap, Vanderbilt University, Nashville, TN, USA) [29,30]. In our reporting, parents and participants with pediatric lived experience are not distinguished and are collectively referred to as family members in the results to protect their privacy.

Due to the focus groups being conducted virtually, participants were required to have an internet connection, have access to an electronic device with a camera, and be proficient in English. To encourage participation in the study, participants were remunerated CA $25 (US $19.39) per hour for their expertise and time. Panels of approximately 3-5 family members or 3-5 clinicians were targeted for each focus group.

### Data Collection

A brief prestudy questionnaire, administered via REDCap [30], collected participants’ demographic information. Next, 2 research team members with expertise in qualitative methods conducted 6 virtual focus groups between December 2020 and August 2021 using Zoom videoconferencing software (Zoom Video Communications, San Jose, CA, USA); 1 researcher facilitated the sessions (author MG or MDW), while another research team member took notes and relayed additional prompts for consideration by the facilitator (author MDW or KC); only the 2 research team members and recruited participants attended each session. Due to potential power dynamics, these sessions were conducted using separate groups for clinicians and family members. At the start of each focus group, each study team member introduced themselves, described their role in the study, and had participants introduce themselves in a similar manner. Next, the facilitator provided a brief overview of our research program, including some background on the use of machine learning in health care and difficulties in communicating procedure-associated risk.

Each focus group session had 2 parts. First, open-ended discussion was structured around 4 themes: (1) how procedure-associated risks (in general) were communicated to families, (2) whether this risk information was clearly understood by families, (3) what tools/methods were typically used to illustrate these risks during the clinical consultation, and (4) whether participants currently used any digital health tools. Second, participants were shown examples of existing risk communication tools [31,32] to elicit preliminary design requirements and visualization preferences to inform prototype development. While viewing examples, participants were prompted to tell researchers their general thoughts on the designs (eg, whether they liked/disliked the design and how these designs could be improved). No repeat interviews were conducted, but we invited participants back for future codesign sessions at the end of each session. Sessions lasted approximately 1 hour, were audio-recorded, and then were digitally transcribed. Participant names were replaced by sequential identifiers, and transcripts were verified by a research team member (KC) rather than participants due to the practicality of conducting sessions online.

### Data Analysis

Focus group transcripts were analyzed using NVivo (QSR International, Melbourne, Australia), and results were summarized using thematic analysis [33]. Two research team members (MDW and KC) independently reviewed 2 transcripts and used inductive coding [34] to organize transcript text by theme, subtheme, and participant type [35]. These researchers then compared interpretations and developed consistent codes. This coding framework was then applied to the remaining 4 transcripts (ie, deductive coding) [34]; however, the 2 researchers discussed any additional themes that emerged after coding these remaining transcripts, resolving any further discrepancies, and inductively modified the coding framework to ensure that key concepts were not missed. Due to the qualitative nature of the study, we did not estimate a target sample size. Alternatively, we implemented a saturation criterion (ie, additional data collection and analysis lead to informational redundancy) [36]; specifically, 2 research team members (MDW and KC) determined that similar comments and concerns were repeatedly discussed across focus groups and that data saturation had occurred.

Finally, prominent themes that emerged from focus groups (see the Results section) were used to generate design requirements for a prototype risk communication tool. Participant responses to the open-ended questions defined when and how our tool would be used and suggested points in the clinical process that need to be addressed and potentially improved, whereas feedback on the sample visualizations provided information to design the prototype for desirability and accessibility. Our prototype was developed using an iterative process in which the research team created, discussed, and revised a preliminary prototype using Figma (Figma Inc, San Francisco, CA, USA) to serve as the baseline for future codesign and pilot evaluation sessions, which may include mixed sessions with clinicians, people with lived surgical experience, and various family members (ie, parents, children, or adolescents).

## Results

### Demographics and Questionnaire Results

In total, 19 participants, including 10 (53%) clinicians (4, 40%, nurse practitioners, 6, 60%, physicians) and 9 (47%) family members, attended 6 focus group sessions with 2-4 participants per session; 4 family members could not be contacted after consenting, 1 declined due to lack of interest and availability, and 1 clinician refused while being approached, due to limited availability. Participants included 15 (79%) females, and 13 (68%) of 19 participants were under 49 years of age. Clinicians worked in anesthesiology and pain management, or surgical/perioperative nursing. Family members included 7 (78%) with either a certificate (university/nonuniversity) or university degree and 2 (22%) with a high school diploma (or equivalent).

### Procedure-Associated Risk Communication in Practice: Key Themes

#### Risk Communication Process Overview

Clinicians indicated they consider risk based on both the patient’s medical history and the specific procedure. Next, they approach family members with a preformulated care plan, which entails discussing the typical patient postoperative experience and any specific concerns that might contribute to increased risk of pain. Clinicians believed it was their responsibility “to try and be unbiased” (clinician 1) and ensure that they “have an honest conversation” (clinician 2) with families to ensure that procedural consent is “not forced upon them in any way” (clinician 2). Clinicians largely did not “mention any of the more severe [or] scary risks that could lead to poor outcomes” (clinician 3) unless it had a high probability of occurrence, it was related to a specific procedure with well-established risks (eg, epidural catheter insertion), or the family had specifically requested further information.

Most family members described a similar risk communication process, felt that the procedure’s associated risks were effectively described, and felt that they were adequately prepared. However, some family members indicated that risk had not been adequately described, for example, in the context of emergency surgery, and 1 indicated that “there's a very standard list of the risks associated with the surgeries that clinicians go through, including pain, but very little discussion around contextualization of these risks” (family member 1).

Hence, we identified a design criterion that risk information should be presented clearly and with appropriate contextualization (requirement R1.1; see Table 1).

**Table 1:** Procedure-associated risk communication in practice and identified design and feature requirements from focus groups with clinicians and family members.

Requirement	Description
R1.1	The risk information should be presented clearly and with appropriate contextualization.
R1.2	Risk information should include a numeric risk score that is contextualized.
R1.3	Preoperative family preparation for their surgical visit should be facilitated by presenting risk information with appropriate checklists.
R1.4	Risk information should be provided using a digital tool to facilitate recall and sharing with other family members.
R1.5	The risk tool should include specific prompts to ensure family member comprehension of the risk information presented.

#### Generalized Risk Statements Used for Clarification

Clinicians explained that they typically describe the severity of a procedure-associated risk descriptively (eg “low,” “moderate,” or “high”) but may provide a numerical representation with a comparative example for contextualization, such as “There is an approximately 1 in 10,000 risk of [a] motor vehicle accident on the way to the hospital, which is similar to the risk of a significant issue or complication with the anesthetic, and that makes it very rare” (clinician 4).

Although families generally agreed that risk was communicated effectively, most had difficulty recalling how risk was specifically conveyed: “I believe [in our initial consultation] the clinician was giving numbers…He may have added, like, a comparison, or an anecdote, but I don't recall any specifics of that” (family member 2). Participants suggested that providing a real-life comparative scenario, such as “winning the CA $2 million jackpot,” would contextualize risk statements and “be easier to remember” (family member 3).

Thus, we identified a design criterion that risk information should include a numeric risk score that is contextualized (requirement R1.2).

#### Methods Used to Communicate Risk in Clinics

Clinicians said that although risk was most frequently explained verbally, some clinicians used whiteboard or paper-and-pencil illustrations, checklists of risks/complications for procedures, existing clinical tools (eg, the Faces Pain Scale), or medical equipment (eg, an epidural catheter) as educational adjuncts to explain aspects of the procedure. When discussing a complex surgical procedure (scoliosis correction), a clinician used “a preprinted list of risks that I go through for every spine patient, which I tick off when I'm seeing the family…I then give the risk checklist to the family, which has the percentage of risk at the top” (clinician 4).

Family members agreed that risk was predominantly communicated verbally and that educational material, such as pamphlets and checklists, provided during the preoperative consultation were informative and, if not lost, could help preparation and stress reduction for the surgical visit.

We identified a design criterion that risk information should provide a preoperative opportunity to help family members prepare for their surgical visit (requirement R1.3).

#### Experience with Health Technology to Communicate Risk

Clinicians stated that they typically do not use health technology to communicate risk information to patients, though some use it for their own learning or when teaching trainees and others. Clinicians also provide preoperative education via locally developed or curated videos about what to expect on the day of surgery. Some family members believed digital communication was “a little bit easier to find, maintain, and store” (family member 2) and reported using smartphone calendars and reminders for appointments and medication adherence.

We identified a design criterion that risk information should be provided as a digital tool (with the option of a hard copy) to facilitate recall and sharing with other family members (requirement R1.4).

#### Family Member Comprehension of Risk

Despite clinicians’ insistence that family members are informed of the risks associated with a procedure, most participants recognized that they were not asked whether they specifically comprehended the risk information presented. As a clinician indicated regarding risk comprehension, “I do not routinely ask patients to repeat back to me what I've said” (clinician 5). Clinicians generally indicated that the last question is always “Do you have any questions?” and that one would assume “if something wasn't understandable to the family, then they would ask at that time” (clinician 4); if there are no further questions, it is assumed that family members adequately understand the given risk(s). As outpatient surgery is common in pediatrics and perioperative discussions are particularly time limited, clinicians highlighted that day surgery visits allow little opportunity to elaborate on risks and resolve questions and indicated that risk communication should ideally occur at a preoperative consultation.

Family members further indicated that contacting staff to answer questions was difficult and often resulted in them using the internet for answers instead; for example, “I was trying to reach the nurses and the hospital clinic and there was no answer, and after 3 days, a nurse called me and then she explained [the discharge instructions]. Other than that, my only help was Google” (family member 4). As the consultation is “meant to inform the patient’s decision of what to expect from the surgery and whether or not to have it” (family member 5), assessing comprehension would allow clinicians “to very quickly help educate and correct any misconceptions [or] to readjust the patient’s understanding of what those risks are” (clinician 6).

We identified a design criterion that the risk tool should include specific prompts for use by both clinicians and family members to ensure comprehension of the risk information presented (requirement R1.5).

### Additional Feature Considerations Indicated from Critically Reviewing Risk Communication Tool Examples

#### Multimodal Presentation of the Risk Score

Participants suggested that risk information should be presented in a multimodal format; this finding was succinctly indicated by a participant that the risk communication tool needs to “maximize the likelihood of finding an approach that any given viewer is going to be able to effectively comprehend” and “accessible and diverse enough in its application to be easy for the clinicians to use as well” (family member 5).

We identified a design criterion that presentation of the risk score should be multimodal and include a simple graphical visualization and that the score should be contextualized with text indicating the percentage and a descriptive risk severity (ie, mild, moderate, or severe) (requirement R2.1; see Table 2).

**Table 2:** Additional design and feature considerations indicated from viewing risk communication tool examples with clinicians and family members.

Requirement	Description
R2.1	Presentation of the risk score should be multimodal and include a simple graphical visualization, contextualized with text, and a descriptive risk severity (ie, mild, moderate, or severe).
R2.2	Color coding should be based on nonthreatening and a color-blindness-friendly palette (ie, shades of blue) to represent severity.
R2.3	Information should be provided about how the risk prediction score was derived by including the most significant factors that contribute to that patient’s level of risk.
R2.4	Risk mitigation strategies should be provided to help family members potentially decrease the patient’s level of risk.

#### Appropriate Color Coding of Risk Visualization

Participants recognized that color coding the score may be problematic for users who are color blind and that, for example, using red may indicate danger/harm to the reader, which might contradict the clinician’s responsibility to communicate “risk in a nonthreatening, nonfrightening way to the family” (clinician 2). However, some clinicians felt that a color, such as red, could be immediately illustrative and attract their attention to modify a patient’s care plan.

Hence, we identified a design criterion that color coding should be based on a nonthreatening and color-blindness-friendly palette (ie, shades of blue) to represent severity as 1 mode of risk presentation (requirement R2.2).

#### Provide Patient Risk Factors and Mitigation Strategies to Allow Agency Over Care

Participants indicated that they would want to see information indicating “what the risk factors actually are and why that patient is high risk” (clinician 7). Clinicians anticipated that clearly identifying these patient risk factors and providing appropriate risk mitigation strategies would give family members “a sense of control, a sense of something to work on to improve their postsurgical outcomes” (clinician 7). Family members acknowledged that having accurate information prior to surgery would make them feel more prepared and would be better than potentially unreliable online resources. Participants suggested that sharing this resource digitally or giving family members a hard copy to take home would improve information accessibility and retention.

We identified a design criterion that the most significant factors that contribute to that patient’s current level of risk should be presented (requirement R2.3). We also identified that risk mitigation strategies should be provided to potentially decrease the patient’s level of risk (requirement R2.4).

### Identified and Implemented Design Requirements Resulting in Prototype Generation

**Figure 1:**
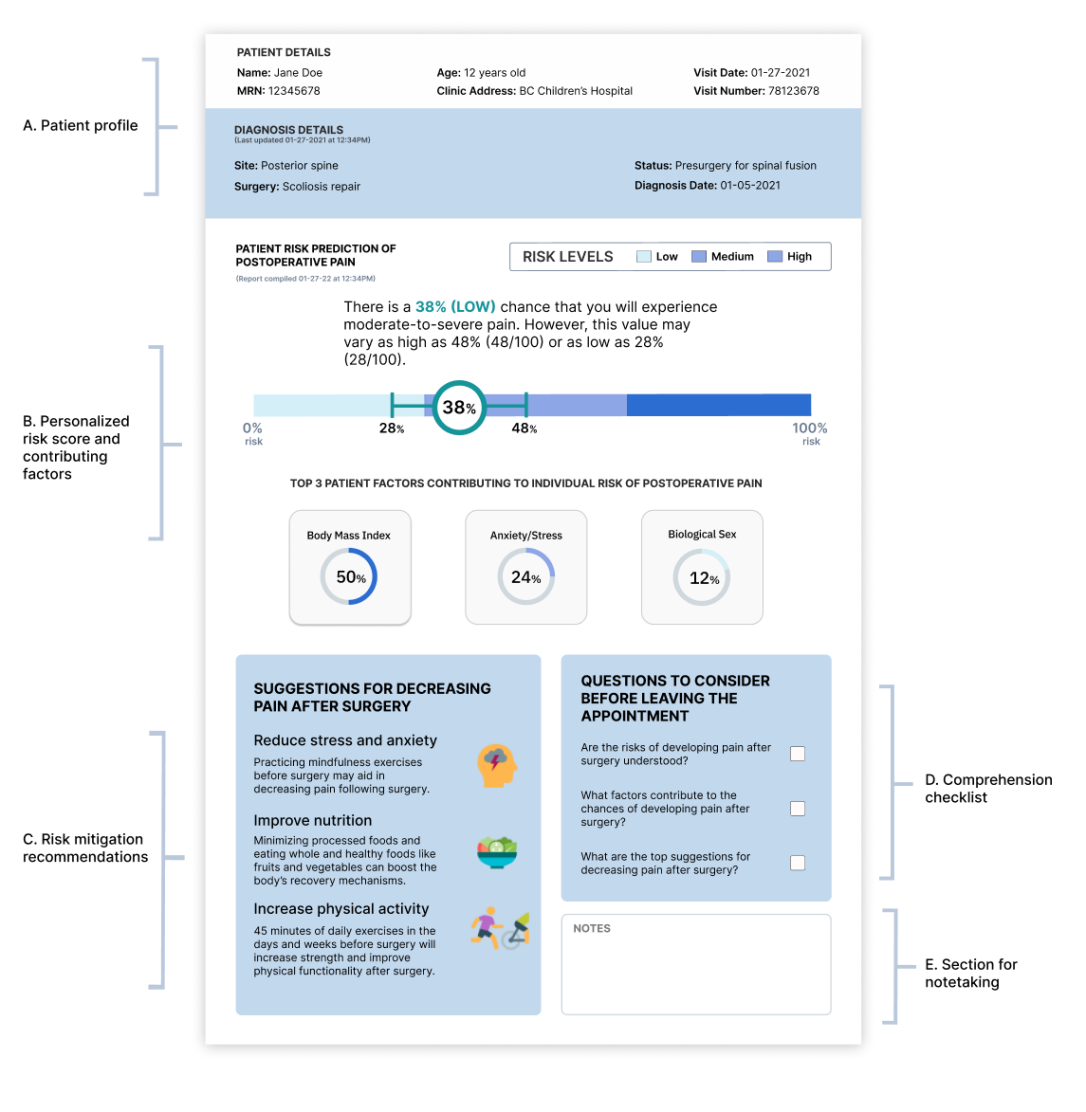
Initial prototype of a potential risk communication tool. (A) Indicates patient demographics and clinical characteristics. (B) Provides a low-medium-high color-coded risk scale with a textual statement and plots to represent the individual’s level of risk and the top factors contributing to the score. (C) Provides mitigation strategies for patients to decrease their chance of pain after surgery. (D) Provides a checklist of questions for patients to consider before leaving their appointment. (E) Provides a blank box for clinicians or family members to take notes during the clinical consultation. BC: British Columbia.

Prominent design and feature requirements that informed the development for our prototype risk communication tool (Figure 1) are summarized in Tables 1 and 2. The initial prototype had 5 sections: (A) demographics and clinical characteristics (not a requirement from the focus groups but included practically to facilitate future implementation in a clinical setting), (B) a color-coded risk scale with a textual statement and plots to present the individual’s level of risk and the top factors contributing to the score (requirements R1.1, R1.2, R2.1, R2.2, and R2.3), (C) mitigation strategies that patients could follow to reduce their risk of postoperative pain (requirements R1.3 and R2.4), (D) a checklist of questions that family members or clinicians can use to ensure that risk information is understood (requirements R1.3 and R1.5), and (E) a section for users to take notes during the consultation to facilitate recall and sharing with other family members (requirement R1.4). The design fits on a traditional 8.5- × 11-inch letter paper for printing but is also suitable for a web-based application and could be adapted for tablets/smartphones (requirement R1.4).

## Discussion

### Principal Findings

Participants indicated that anesthetic and pain risk is typically communicated verbally to patients and their family members, with its severity expressed descriptively or numerically or in both ways, which may be expanded upon with a comparative example for contextualization. It was deemed imperative that family members be provided with risk information and then allowed time to reflect and follow up with questions or concerns. Participants specified the following key design requirements and feature considerations: (1) present risk information clearly and with contextualization, (2) quantify the risk and contextualize it, (3) include checklists for preoperative family member preparation, (4) provide risk information digitally to facilitate recall and sharing, (5) query the family member’s understanding to ensure comprehension of risk, (6) present the risk score using multimodal formats, (7) use color coding that is nonthreatening and avoids limitations with color blindness, (8) present the most significant factors contributing to the risk prediction score, and (9) provide risk mitigation strategies to potentially decrease the patient’s level of risk. Our initial risk communication tool prototype embodies all identified requirements and features.

### Comparison With Prior Work

Using iterative feedback from patient partners and a multidisciplinary team of clinicians, researchers at the Ottawa Hospital developed the Personalized Risk Evaluation and Decision Making in Preoperative Clinical Assessment (PREDICT) risk score [37]; this tool generates a multimodal risk analysis composed of numerical absolute risks, a pictograph, brief contextual statements, and guiding questions to encourage discussion of their care and facilitate shared decision-making [37]. Importantly, participants using the PREDICT app had significantly better knowledge of their risk profile, reported lower anxiety, and reported higher satisfaction scores relative to the standard of care, and no surgeries were cancelled as a result of exposure to the risk score [37]. This suggests that communicating individualized risk in a clear and concise multimodal format has the potential to improve clinically relevant outcomes and ensure that patients are informed of procedure-associated risks. Although the PREDICT app had substantially different outcome assessments (ie, morbidity, mortality, and expected length of stay) and targeted adults, compared to our proposed pediatric risk score (ie, postoperative pain), focus group participants indicated similar design requirements. The PREDICT app communicated risks as population-informed personalized risks (eg, “For people like you who had this surgery, 10 of 100 had a serious complication postsurgery.”) [37], whereas our prototype indicates the individual’s risk (ie, “There is a 38% chance your child will experience moderate-to-severe postoperative pain.”). As such, future iterations of our prototype may need to assess which phrasing choices end users prefer to effectively indicate their level of risk.

A study from the University of Toronto used semistructured interviews with end users and key stakeholders to establish design requirements for a risk communication tool to predict radiation toxicity risk for patient with cancer using machine learning [32]. Their user interface requirements included patient information, variables associated with risk prediction, prediction accuracy, integration of user feedback into the tool, links to validation studies, the outcome’s expected time frame (eg, risk in the next 30 days), and a graph of risk over time [32]. Our prototype includes similar design requirements; yet, the Toronto team identified 2 additional requirements to consider: (1) indicating changes in the predicted risk over time and overlaying this information with clinical events, which has the potential to both illustrate the clinical impact that tailored risk reduction strategies have on a patient’s level of risk and provide insight into why that risk increased/decreased, and (2) including a feedback mechanism in the application’s user interface to assess the agreement between clinical judgment and tool prediction/recommendation (ie, “Do you agree with this prediction? Did you follow the recommendation?”) [32]. This feature may enable early assessment of any discrepancies between our model’s prediction and the clinical utility of resulting recommendations and, hence, may be a useful addition to our risk communication tool.

A recent study surveyed communication needs and preferences of pediatric patient families and indicated that their primary preoperative concern was complications/risks associated with the procedure/treatment, which highlights the importance of effective risk communication [38]. Verbal communication was the preferred modality, but many families indicated that a list of complications, percentages, and diagrams were also desirable [38]. Although our prototype includes a multimodal risk score, clinicians should present the risk score to patients and their family members. Families also prefer to resolve queries following discharge over the telephone, a short message service app, or email, and the most important element of a “good” perioperative experience is effective communication with the health care team [38]. Thus, implementing communication features in our risk tool may be necessary for successful implementation.

Finally, pain risk communication may have significant nocebo effects (ie, where unintended negative suggestions/phrasing about a treatment/procedure result in increased adverse events) [39], such as loss of appetite, nausea, itching, and stomach pain [40]. Phraseology is important [39], and framing an opportunity to improve future patient comfort by identifying relevant and modifiable risk factors, instead of highlighting risks of pain and unmodifiable risk factors, might reduce these potential nocebo effects. As 1 of the design requirements of our prototype is to present risk in a nonthreatening manner, we may wish to limit the use of the initial prototype to clinicians, while developing a version that focuses on “optimizing comfort” rather than “reducing pain” for sharing with family members. Similar to the PREDICT app [37], we should consider tracking child and parent anxiety levels, user satisfaction, and surgery cancelations when our tool is used to confirm that its presentation does not result in unintended consequences that could impede recovery following surgery.

### Limitations

Our clinician participants comprised a relatively small cohort of anesthesiologists and nurse practitioners, which represents a sampling bias [41] that may limit the transferability (ie, external validity) [42] of our findings to other hospital sites and settings, as well as to other health care professions. As such, a larger and more diverse cohort of health care team members (eg, surgeons, physiotherapists, psychologists, and medical office assistants), and family members, may be desirable in future studies; we plan to recruit a wider range of health care workers for our codesign and pilot evaluation sessions. As children have the right to acquire information pertinent to their health and well-being [43], it may also be imperative to include children over 7 years old in future sessions to facilitate future implementation in pediatric care. Next, our focus groups comprised only English-speaking participants, which may have further limited transferability; language interpretation services and closed captioning (when virtual) were offered during recruitment but will be highlighted for future sessions. Although focus groups were conducted virtually, our sample may not be representative of harder-to-reach communities, and our tool may lack some requirements for effectively communicating risk with them. Furthermore, our focus groups comprised separate cohorts of clinicians in one set of meetings and family members in another set, which limited interactions among participants. Due to the potential power imbalance between family members and clinicians, we decided to conduct these initial focus groups separately. Given the users’ previous education level, the risk score may be difficult to interpret and may not clearly guide clinical decision-making, but this was beyond the scope of our current study. Lastly, our risk score prediction statements and interpretations (eg, 38% chance of moderate-to-severe pain following surgery) were generated by the research team from the identified requirements as examples and do not represent definitive interpretations of these concepts. Although participants did not provide feedback on our current findings, we plan to conduct mixed group codesign workshops to further develop the prototype and obtain qualitative feedback on the tool prior to usability evaluation.

### Conclusion

Our study identified several design requirements for personalized risk communication, such as presenting risk in a nonthreatening/nonfrightening manner; providing a comprehensive multimodal format, including top contributing variables to the pain risk score; providing a comprehension checklist; and providing potential risk reduction strategies. Although further family-centered design and clinical evaluation are needed, we envision that implementing a risk communication tool into clinical practice has the potential to bridge existing gaps in the accessibility, utilization, and comprehension of personalized risk information between health care professionals and family members of pediatric surgical patients.

## Abbreviations

BCCH: British Columbia Children’s Hospital
PREDICT: Personalized Risk Evaluation and Decision Making in Preoperative Clinical Assessment
REDCap: Research Electronic Data Capture
